# Efficacy of a Multimodal Ayurveda Regimen in the Management of Primary Knee Osteoarthritis: Protocol for an Open-Label Randomized Controlled Trial

**DOI:** 10.2196/68306

**Published:** 2025-09-03

**Authors:** Amit Kumar Rai, Babita Yadav, Uma Kumar, Bharti Gupta, Kishore Patel, Shruti Khanduri, Bhagwan S Sharma, Richa Singhal, Bhogavalli Chandrasekhararao, Narayanam Srikanth, Rabinarayan Acharya

**Affiliations:** 1 Department of Kayachikitsa Ayurvedic and Unani Tibbia College and Hospital New Delhi India; 2 Department of Ayurveda Central Ayurveda Research Institute New Delhi India; 3 Department of Rheumatology All India Institute of Medical Sciences New Delhi India; 4 Department of Ayurveda Central Council for Research in Ayurvedic Sciences Ministry of Ayush, Government of India New Delhi India; 5 ICMR-National Institute of Malaria Research, New Delhi New Delhi India; 6 Central Council for Research in Ayurvedic Sciences Ministry of Ayush, Government of India New Delhi India

**Keywords:** Janu Basti, Laksha Guggulu, Matra Basti, osteoarthritis, WOMAC, Western Ontario and McMaster Universities Osteoarthritis Index

## Abstract

**Background:**

Ayurveda recommends a comprehensive therapeutic approach for osteoarthritis management. However, most of the published clinical studies on Ayurveda interventions for osteoarthritis management have addressed selected modalities of Ayurveda treatment rather than the holistic therapeutic regimen.

**Objective:**

This study aimed to assess the efficacy and safety of a multimodal Ayurveda treatment protocol in the long-term management of primary osteoarthritis of the knee compared with standard care.

**Methods:**

The proposed open-label, parallel-group randomized controlled trial was conducted in individuals of any gender aged 40 to 70 years and diagnosed with primary osteoarthritis of the knee as per the American College of Rheumatology criteria. Individuals with grade 4 radiographic changes in the affected knee (based on the Kellgren-Lawrence classification) and with comorbidities were not considered. The study was conducted at the All India Institute of Medical Sciences, New Delhi, India. A total of 150 participants underwent random assignment in a 1:1 ratio to receive either the Ayurveda treatment protocol or conventional standard care for 180 days. The primary outcome was the change in the Western Ontario and McMaster Universities Osteoarthritis Index score from baseline. The secondary outcome measures included the change in the score for pain, stiffness, and physical function from baseline; change in the range of motion of the knee joint; change in the score of the numeric pain rating scale, Pain Disability Index, and 12-item short-form survey questionnaire (to assess health-related quality of life); change in highly sensitive C-reactive protein levels, interleukin-6 levels, magnetic resonance imaging scan findings, and dual-energy X-ray absorptiometry scan findings; and change in the need for rescue analgesic medication from baseline. Safety was evaluated by recording the incidence of adverse events and changes in liver and kidney function tests from baseline.

**Results:**

Recruitment of study participants commenced on October 11, 2022. Currently, all the participants completed the study and the analysis of the study outcomes is ongoing.

**Conclusions:**

This randomized controlled trial will be the first study to explore the potential benefits of a multimodal Ayurveda regimen (based on Ayurveda principles and scientific evidence) in the long-term management of osteoarthritis of the knee through validated subjective, laboratory, and imaging parameters. The outcomes of this study may address the needs and challenges associated with osteoarthritis management.

**Trial Registration:**

Clinical Trial Registry of India CTRI/2022/05/042792; https://ctri.nic.in/Clinicaltrials/pmaindet2.php?EncHid=Njk0MTM=&Enc=&userName=

**International Registered Report Identifier (IRRID):**

DERR1-10.2196/68306

## Introduction

### Background

Osteoarthritis is a chronic degenerative condition characterized by structural changes in the articular cartilage, ligaments, capsule, synovium, subchondral bone, and periarticular muscles, resulting in joint pain, stiffness, and limited mobility [1]. As per the Osteoarthritis Research Society International, osteoarthritis has all the hallmarks of a serious condition because of associated disability and loss of function [2]. Osteoarthritis most commonly affects the knee, followed by the hand, hip, spine, and shoulder. Osteoarthritis of the knee accounts for approximately 85% of osteoarthritis cases globally [3]. Primary osteoarthritis is caused by age-related degeneration, while secondary osteoarthritis occurs because of injury, occupation-related stress on joints, and comorbid conditions, including diabetes mellitus. Mechanical, inflammatory, and metabolic factors play an important part in the intricate pathophysiology of osteoarthritis, which eventually results in the structural breakdown and collapse of the synovial joint [4]. Global estimates indicate that approximately 595 million individuals worldwide were affected by this burdensome clinical condition in 2020, equivalent to 7.6% of the global population [5]. Osteoarthritis is becoming more prevalent owing to the combined impacts of aging, rising physical inactivity and obesity in the population, and an increased incidence of joint injuries [2]. In 2020, 21.7 million years lived with disability were attributed to osteoarthritis globally [5]. It is one of the 4 leading causes of years lived with disability worldwide [4]. The objectives of osteoarthritis management are to reduce pain intensity and inflammation, slow down cartilage degradation, improve joint movement, and reduce disability. Treatment options include patient education, oral and topical pharmacological agents, interventional procedures (eg, corticosteroid injection and viscosupplementation), bracing, assistive devices, physical therapy, and surgery (eg, arthroscopy, osteotomy, and arthroplasty) [6]. Currently, conventional medicine has no therapeutic options that can arrest or slow down the disease progression in patients with osteoarthritis [2]. In the absence of drugs with proven disease-modifying activity, the current approach is mainly focused on palliative pain management [7]. However, chronic pain management with acetaminophen, nonsteroidal anti-inflammatory drugs, and intermittent intra-articular corticosteroid injections is associated with limited benefits, along with considerable gastrointestinal, renal, and cardiovascular risks [2,8-10]. Furthermore, there is uncertainty regarding the effect of glucosamine and chondroitin supplementation on the structural progression of osteoarthritis [10]. Meanwhile, arthroplasty is considered only for severe cases of osteoarthritis. Therefore, it is pertinent to search for safe, efficacious, and cost-effective therapeutic options with better tolerability to reduce the disease burden of osteoarthritis.

*Sandhigatavata*, a specific type of *Vatavyadhi* (disease conditions because of vitiation of *Vata Dosha*) in Ayurveda, has a clinical presentation similar to osteoarthritis. Ayurveda suggests a multimodal treatment approach for managing *Sandhigatavata*, a traditional Ayurvedic condition clinically comparable to osteoarthritis, including a judicious combination of *Panchkarma* (Ayurveda therapeutic procedures), oral medications, nutritional supplements, a nutritious diet, and a recommended lifestyle. Several published clinical studies have highlighted the safety and beneficial effects of Ayurveda interventions in the management of osteoarthritis [11-23]. One randomized controlled trial (RCT) concluded that the Ayurveda treatment protocol led to significant and clinically relevant improvement in osteoarthritis-related clinical features compared with conventional care, with most effects maintained over 12 months [11,12]. Another clinical study, supported by the World Health Organization to evaluate the feasibility of operational integration of Ayurveda treatment with conventional medicine in the management of osteoarthritis of the knee at a tertiary care hospital, also reported that the Ayurveda treatment regimen showed promising outcomes in the management of osteoarthritis of the knee, including symptom reduction, improved quality of life, and reduced need for conventional analgesics [13]. A systematic review also highlighted the safety and preliminary evidence regarding the effectiveness of various Ayurveda interventions in the management of osteoarthritis [24]. In addition, Ayurveda interventions, such as *Shallaki* (*Boswellia serrata* Roxb.)*, Guggulu* (*Commiphora mukul* Engl.)*, Ashwagandha* (*Withania somnifera* [L.] Dunal)*, Shunthi* (*Zingiber officinale* Roscoe)*, Bala* (*Sida cordifolia* L.)*, Nirgundi* (*Vitex negundo* L.)*,* and *Dashmula* (a combination of the dried root of 10 specific medicinal plants) have shown potential anti-inflammatory, analgesic, antiosteoporotic, and antioxidant activities [25-33].

However, most of the aforementioned published studies have addressed selected modalities of Ayurveda treatment rather than a holistic Ayurveda therapeutic regimen. Therefore, it is necessary to conduct RCTs with a multimodal Ayurveda regimen for the management of osteoarthritis. Considering this limitation, the present RCT was conceptualized to assess the efficacy and safety of an Ayurveda treatment regimen (consisting of therapeutic procedures and oral medications) to manage primary osteoarthritis of the knee. The therapeutic regimen proposed for the study is based on preliminary leads from exploratory clinical studies on individual Ayurveda interventions that have shown promising outcomes in the management of osteoarthritis [15,17,18].

### Objectives

The present RCT was designed to assess the efficacy of multimodal Ayurveda interventions in managing pain and functional disability in patients with primary osteoarthritis of the knee compared with standard care. The key secondary objectives of this study included evaluating the efficacy of the trial Ayurveda interventions on range of motion of the knee joint, the need for rescue analgesic medication, quality of life parameters, knee structural changes (ie, delay in the structural progression of osteoarthritis), proinflammatory biomarkers, and bone mineral density after 180 days. Another secondary objective was to assess the safety of multimodal Ayurveda interventions in managing osteoarthritis of the knee.

## Methods

The study protocol has been drafted following the SPIRIT (Standard Protocol Items: Recommendations for Interventional Trials) guidelines [34].

### Study Design and Setting

This clinical study was an open-label, randomized, controlled, parallel-group, noninferiority trial. The study was conducted at the All India Institute of Medical Sciences (AIIMS), New Delhi, India.

### Study Participants

Individuals of any gender aged 40 to 70 years who were diagnosed with primary osteoarthritis of the knee as per the American College of Rheumatology (ACR) criteria; had a pain score of at least 2 (as per the numeric pain rating scale) on most days in the last month; met ACR functional status criteria class I, II, and III; have a serum vitamin D level >30 ng/mL; and were willing to provide written informed consent were included in the study. The ACR criteria for osteoarthritis of the knee include knee pain along with any 3 of the following: aged >50 years, joint stiffness lasting less than 30 minutes, crepitus, bony tenderness, bony enlargement, and absence of palpable warmth.

Individuals were excluded if they had grade 4 radiographic changes in the affected knee joint (as per the Kellgren-Lawrence classification); had a history of significant trauma to the knee (including arthroscopy within the preceding year), knee joint replacement, intra-articular corticosteroid or hyaluronic acid administration within 1 month before the study; were currently taking corticosteroids; or had a history of rheumatoid arthritis, psoriatic arthritis, systemic lupus erythematosus, cardiovascular disease, or malignancy. Individuals were also excluded if they had uncontrolled diabetes mellitus (ie, hemoglobin A_1c_ >8%), uncontrolled hypertension (ie, blood pressure >160/100 mm Hg despite medication), abnormal hepatic function (ie, aspartate aminotransferase or alanine aminotransferase >2 times the upper limit of normal), or abnormal renal function (ie, serum creatinine >1.2 mg/dL); a tendency for occurrence of recurrent renal calculi; or a BMI ≥32 kg/m^2^. Similarly, those with any contraindication for magnetic resonance imaging (MRI), a history of hypersensitivity to the study interventions, a history of chain smoking, alcohol use disorder, substance abuse, or any other clinical condition that the investigator believed may compromise the participant’s safety, compliance, or evaluation were not considered for the study.

### Study Intervention

Eligible participants in the Ayurveda group received a comprehensive Ayurveda treatment protocol, which included *Matra Basti* with *Ksheerabala Taila* and *Janu Basti* with *Dhanwantara Taila*, along with oral medications—*Laksha Guggulu* and *Muktashukti Bhasma*—for 180 days. The details of the study interventions are provided in Table 1.

**Table 1:** Details of the study interventions.

Intervention (with reference)	Route of administration	Dosage and frequency	*Anupana* (vehicle of administration)	Duration
*Ksheerabala Taila* (API^a^ part II, vol I [35])	Rectal (*Matra Basti*)	60 mL once daily after food	—^b^	14 d every 2 mo for 180 d
*Dhanwantara Taila* (API part II, vol I [35])	Local application over the affected knee (*Janu Basti*)	QS^c^ once daily	—	14 d every 2 mo for 180 d
*Laksha Guggulu* (API part II, vol II [36])	Oral	1 g twice daily after food	Lukewarm water	180 d
*Muktashukti Bhasma* (Pharmacopeial Standards for Ayurvedic Formulations [37])	Oral	250 mg twice daily after food	Lukewarm water	180 d

^a^API: Ayurvedic Pharmacopoeia of India.

^b^Not applicable.

^c^QS: quantity sufficient.

The methodology of the therapeutic procedures, *Matra Basti* and *Janu Basti*, is provided in Multimedia Appendix 1. The participants in the control group will receive standard care (ie, topical application of diclofenac sodium gel twice daily) for 180 days, as recommended in the standard treatment guidelines for the management of osteoarthritis of the knee, issued by the Ministry of Health and Family Welfare, Government of India.

Ayurveda practitioners on the research team dispensed the Ayurveda interventions at the study sites throughout the trial period. Furthermore, paramedical staff trained in *Panchakarma* administered *Matra Basti* and *Janu Basti* to the study participants in the Ayurveda group. The participants were examined every 30 days during the study period. The trial Ayurveda interventions were procured from the Indian Medicines Pharmaceutical Corporation Limited, Ministry of Ayush, Government of India.

### Discontinuation of the Study Interventions

The study interventions are not causally associated with serious adverse effects or adverse drug reactions (ADRs), as reported in previously published studies. However, if appropriate measures are not followed while administering *Janu Basti*, adverse effects, such as mild irritation or skin rashes, may occur at the application site. Similarly, bloating, increased bowel movements, and abdominal pain may develop if *Matra Basti* is not administered correctly. *Laksha Guggulu* may lead to abdominal discomfort, nausea, belching, and skin rashes if not taken as per the physician’s instructions and recommended dose.

If any participant developed any adverse effects, administration of the study interventions was temporarily discontinued, and the participant was closely monitored. If symptoms recurred after reintroducing the study interventions, the participant was withdrawn and provided with appropriate medical care as needed.

If any participant developed a serious adverse event (AE) or treatment-emergent AE during the study period, the participant was withdrawn from the study and provided with appropriate incidental care at the study site (ie, the AIIMS, a tertiary care medical institution). In addition, all the study participants were covered by clinical trial insurance during their participation for medical expenses related to the management of any study-related AEs. Furthermore, the sponsor and the ethics committee were notified within 2 working days using the AE and ADRs reporting format, along with appropriate justification. Causality assessment of all AEs, serious AEs, and ADRs occurred during the study was also conducted.

### Compliance With Trial Interventions During the Study

All participants in the Ayurveda group were provided with an information leaflet containing instructions for the use (dose, frequency, and time of administration) and storage of the study interventions. The participants were also issued a compliance form during the baseline and subsequent follow-up visits to self-report their consistent or irregular use of the trial interventions and to record any missed doses with remarks for missing, which enabled assessment of adherence to the dosing pattern as per the study protocol. During each follow-up visit, participants were asked to return the used, unused, or partially used containers of the study interventions to the investigators to assess adherence and cross-check with the participant’s self-reported compliance form.

Participants who did not adhere to the study protocol, did not have ≥80% compliance, developed any study-related AEs resulting in withdrawal from the study at their preference, or withdrew their voluntary consent for participation in the study were withdrawn from the study. If participants consented to data collection during the scheduled follow-ups or at the end of the 180-day period, the data were collected and recorded in the case record form (CRF). The data from completed assessments available up to the point of withdrawal will be used for analysis.

### Concomitant or Rescue Medication

The investigators monitored the participants for any concomitant or rescue medication required during the study period. In the event of any AEs or an increase in pain intensity, rescue medication was permitted at the investigators’ discretion. All instances of concomitant care were carefully documented in the CRF.

### Outcome Measures

The primary outcome was the change in the Western Ontario and McMaster Universities Osteoarthritis Index (WOMAC) score from baseline. The secondary outcome measures included the change in the score for pain, stiffness, and physical function from baseline (assessed using the WOMAC scale); the change in range of motion of the knee joint (assessed using a goniometer); the change in the scores of the numeric pain rating scale, Pain Disability Index, and 12-item short-form survey questionnaire (to assess health-related quality of life); the change in high-sensitivity C-reactive protein and interleukin-6 levels, MRI findings (to assess knee structural changes), and dual-energy x-ray absorptiometry scan findings (to assess change in bone mineral density); and the change in the need for rescue analgesic medication.

The outcomes were evaluated every 30 days until day 180 from baseline. Laboratory investigations were conducted at baseline and on day 180.

### Safety Assessment

The safety of the trial interventions was determined by recording the incidence of AEs, if any, during scheduled follow-up visits using a structured format. All AEs during the study were recorded and monitored in accordance with the International Council for Harmonisation–Good Clinical Practice (GCP) guidelines. Safety was also evaluated by performing liver function tests (LFTs) and kidney function tests (KFTs) on days 90 and 180 from baseline.

### Sample Size

On the basis of the results from a previous study [11], in which the difference in WOMAC score between the Ayurveda and conventional group after 12 weeks of treatment was 24 points, this study has been designed to have 80% statistical power to detect a difference of at least a 20-point improvement (change from baseline) in the WOMAC score after treatment between the 2 groups (pooled SD 42; 2-sided *t* test, α=.05). To achieve this, 69 participants per group were required. Accounting for an expected attrition rate of 10%, the final sample size for each group was determined to be 75 participants (150 in total).

### Recruitment of Study Participants

**Figure 1:**
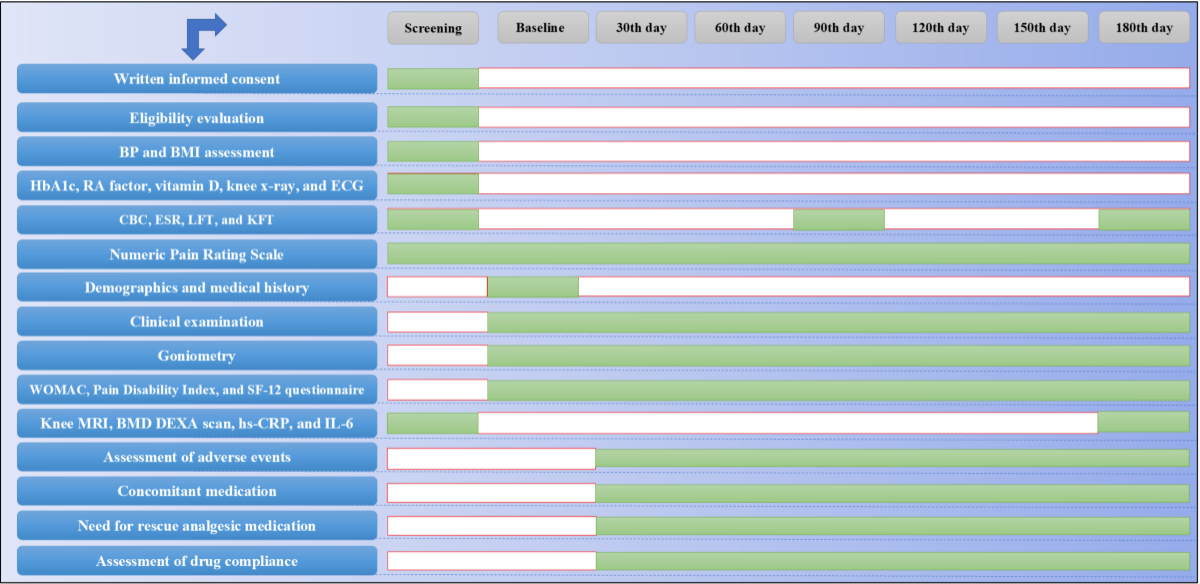
The study schedule. BMD: bone mineral density; BP: blood pressure; CBC: complete blood count; CRF: case record form; DEXA: dual-energy x-ray absorptiometry; ECG: electrocardiogram; ESR: erythrocyte sedimentation rate; HbA1c: hemoglobin A1c; hs-CRP: high-sensitivity C-reactive protein; IL-6: interleukin 6; KFT: kidney function test; LFT: liver function test; MRI: magnetic resonance imaging; RA: rheumatoid arthritis; SF-12: 12-item short-form survey; WOMAC: Western Ontario and McMaster Universities Osteoarthritis Index.

During the study, the investigators screened individuals with clinical features of osteoarthritis of the knee who visited the outpatient department of the study sites, based on the defined inclusion and exclusion criteria, to identify and recruit potential individuals. The research personnel allocated the eligible participants to either of the 2 groups based on a computer-generated randomization schedule (Figure 1). The screening process was continued until the target sample size for the study was achieved.

### Randomization

The eligible individuals were randomly assigned to the Ayurveda group or the control group with a 1:1 allocation. SPSS software (version 15.0; IBM Corp) was used to generate the random number sequences. The randomization sequence was generated by an independent statistician who was not involved in participants’ enrollment and assessment.

### Allocation Concealment

Sequentially numbered, opaque, sealed envelopes were used to ensure allocation concealment. The participant’s enrollment number was printed on the top of the envelope, and a slip indicating the participant’s allocated group was placed inside. After completing all baseline assessments, the research staff provided the sealed envelope to the eligible individuals. The individual opened the envelope and was then allocated to a group as indicated on the slip inside. The opened envelope and the printed slip were attached to the participant’s CRF for documentation and trial monitoring.

### Data Collection

The baseline demographics, clinical, and physical examination data were collected by qualified Ayurveda study personnel and recorded in a CRF designed for this purpose. The subjective and objective outcome assessments were conducted according to the study protocol. Knee pain, stiffness, and functional disability were assessed using the WOMAC score [38]. The self-reported severity of pain was recorded using a numeric pain rating scale and Pain Disability Index [39,40]. The overall quality of life was assessed using a validated scale, the 12-item short-form survey questionnaire [41]. These outcomes were assessed at baseline and during follow-up visits on days 30, 60, 90, 120, 150, and 180.

Serum samples for the objective assessment parameters, such as proinflammatory biomarkers, LFT, and KFT, were collected at baseline and on day 180 (LFT and KFT were also assessed on day 90), and transported to the NABL-accredited laboratory, along with data from MRI and dual-energy X-ray absorptiometry scans. The data received from the laboratory were entered in the CRF and e-CRF. The research team underwent training on the study protocol and standard operating procedures for the study conduct, storage and dispensing of study interventions, handling of biological samples, data collection, and data recording to ensure compliance with GCP principles, while ensuring participant safety, data accuracy, and reliability.

### Data Management

Data management in this clinical study adhered to stringent guidelines to ensure the accuracy, reliability, and integrity of the collected information. Upon the participant’s assessment, the research team promptly entered the data into CRFs and e-CRFs. Source documents and CRFs were securely stored in restricted-access areas, limited solely to the study team. Electronic CRFs were password-protected and stored in secure, access-restricted computer systems.

The data entered by the study personnel underwent meticulous cross-verification by the study investigators at the study site, ensuring the reliability of the data. Rigorous quality measures were implemented, such as regular audits, to identify and address any discrepancies in the data. Data management practices adhered to regulatory guidelines and ethical principles, prioritizing the protection of participant rights.

### Statistical Methods

Categorical data will be presented as numbers (percentages) and compared between groups using the chi-square test. Continuous data following normal distributions will be reported as mean (SD), and between-group comparisons will be performed using the independent sample *t* test. Within-group comparisons for normally distributed data will be conducted using the paired sample *t* test or repeated measures ANOVA. Nonnormally distributed data will be reported as median (first and third quartiles), and between-group comparisons will be performed using the Mann-Whitney *U* test. Within-group comparisons will be conducted using the Wilcoxon signed rank test or the Friedman test. A *P* value <.05 will be considered statistically significant. SPSS software (version 29.0; IBM Corp) will be used for statistical analysis.

The modified intention-to-treat analysis approach will be applied to handle missing data. Missing data for all participants with available data from at least 1 visit after baseline (day 30) will be imputed. The last observation carried forward method will be used to impute the missing values.

### Monitoring

The data and safety monitoring board monitored the study for quality and regulatory compliance. The data and safety monitoring board reviewed the progress of the study every 6 months until the end of the study period.

### Trial Audit

An on-site monitoring visit by an independent committee constituted by the sponsor was planned to ensure that the study procedures and data collection processes complied with existing regulatory standards and to check the accuracy, completeness, legibility, and timeliness of the reported study data.

### Ethical Considerations

The institutional ethics committee of AIIMS, New Delhi, has approved the study protocol (IEC-132/04.02.2022) and related documents to ensure compliance with ethical standards and safeguard the rights and well-being of the participants. The study has been registered prospectively at the Clinical Trial Registry of India (CTRI/2022/05/042792). The study was undertaken in accordance with the principles of the Declaration of Helsinki, the Indian Council of Medical Research’s National Ethical Guidelines for Biomedical and Health Research on Human Participants (2017), and the International Council for Harmonisation GCP guidelines. All substantial amendments to the study protocol affecting participant safety or study integrity were submitted to the institutional ethics committee for approval before implementation. Before undergoing any study-related procedure, potential participants received a participant information sheet in Hindi or their native language. The participant information sheet comprehensively outlined the various aspects of the study, equipping the participants with the necessary information to make an informed decision regarding participation in the study. Written informed consent was obtained using the consent form, signed by the participant and the study personnel delegated to the task.

All relevant study data were securely stored at the study site in password-protected access systems located in areas with limited access. To maintain participant confidentiality, a coded enrollment identification number was used to identify all laboratory specimens, reports, data collection, and relevant forms. All records containing names or other personal identifiers, such as informed consent forms, were stored separately from the study records, which were identified by code identification numbers, in a restricted-access area.

### Ancillary and Posttrial Care

No ancillary studies are proposed in the present clinical study. If required, the participants were provided with routine medical care after completing the study.

## Results

The recruitment of study participants was initiated on October 11, 2022. The recruitment of study participants has been completed. The analysis of the study data is in progress.

## Discussion

### Anticipated Findings

Osteoarthritis, owing to its chronic nature, is associated with considerable disease burden, functional disability, and health care costs globally. Because of inadequate relief and adverse effects associated with conventional medications, a substantial proportion of individuals affected by osteoarthritis prefer traditional medicine systems, such as Ayurveda, for sustained long-term relief without treatment-related safety concerns. Ayurveda adopts a comprehensive treatment approach combining various therapeutic options, such as *Abhyanga* (massage), *Swedana* (hot fomentation), *Mridu Samshodhana* (mild purgation), *Basti* (medicated enema), *Janu-Kati-Greeva-Pristhta Basti* (localized oleation therapy), *Upanaha* (poultice application), *Vatashamaka* (pacifying vitiated *Vata Dosha*), and *Rasayana* (Ayurveda nutritional supplements), along with a nutritious diet and a balanced lifestyle, for the effective management of osteoarthritis depending on the stage of the disease. Numerous clinical studies have been conducted to evaluate the efficacy of various Ayurvedic interventions for managing osteoarthritis. Most of these studies have focused on single herbs, compound formulations, or individual therapeutic procedures. Only a few studies have investigated multimodal therapeutic regimens grounded in Ayurveda principles and practiced in routine Ayurvedic care.

The present RCT was conceived to evaluate the efficacy of an Ayurveda treatment regimen comprising *Panchkarma* procedures (*Matra Basti* and *Janu Basti*) along with oral medications—*Laksha Guggulu* and *Muktashukti Bhasma*—for the management of primary osteoarthritis of the knee. *Matra Basti* (therapeutic enema with medicated oil) using *Ksheerabala Taila* is planned for this study, as *Basti* is considered the prime treatment modality for *Vatavyadhi* (musculoskeletal, neuromuscular, and degenerative disorders) [42]. Furthermore, it is well-established that drug administration via the rectal route can achieve significantly higher blood levels of the medication compared with the oral route, as the rectum has a rich blood and lymph supply; thus, lipid-soluble drugs can cross the rectal mucosa, be readily absorbed, and enter the systemic circulation [17,43]. Thus, the administration of drugs in the *Basti* form may result in faster absorption and quicker results. Bala, the principal constituent of *Ksheerabala Taila*, possesses anti-inflammatory, analgesic, and antioxidant properties [44-46]. In addition, published clinical studies on *Matra Basti* in osteoarthritis have shown promising outcomes [15,17]. Similar results were also reported in an exploratory clinical study on *Basti* with *Ksheerabala Taila* in patients with osteoarthritis of the knee [21]. *Janu Basti* (medicated oil retention over the knee) is a localized Ayurveda procedure used for knee joint–related ailments. During the procedure, warm medicated oil is applied to the knee joint, allowing it to be absorbed into the joint tissues, thereby providing lubrication and improving joint mobility by enhancing blood circulation and promoting muscle relaxation in the knee area. It also alleviates pain and inflammation through the anti-inflammatory and analgesic properties of the ingredients of the medicated oil. Published clinical studies and case reports have reported the potential benefits of *Janu Basti* in providing relief for osteoarthritis-related outcome parameters [47-49]. Furthermore, in this study, *Dhanwantara Taila* is proposed for *Janu Basti*, which has significantly relieved osteoarthritis-related complaints and improved the quality of life and WOMAC score in previous research studies [20]. In addition, the major ingredients of *Dhanwantara Taila*, namely, *Bala* and *Dashamula*, possess analgesic and anti-inflammatory properties [33,44-46]. An experimental study also reported the anti-inflammatory and analgesic activities of *Dhanwantara Taila* [50].

The ingredients of *Laksha Guggulu* possess anti-inflammatory, analgesic, antiarthritic, chondroprotective, and antioxidant activities, and therefore are likely to ameliorate disease progression in patients with osteoarthritis [27,28,51-54]. In addition, an experimental study highlighted the antiarthritic and chondroprotective potential of *Laksha Guggulu* [55]. Exploratory studies on *Laksha Guggulu* have provided preliminary evidence of its efficacy in osteoarthritis of the knee and osteoporosis [18,56,57]. *Muktashukti Bhasma* is a natural calcium-containing Ayurveda formulation used in degenerative conditions of bones and joints to nourish these tissues. A published standardization study reported it as nano-range calcium carbonate in calcite form [58]. The study also highlighted it as a good source of elemental calcium, as observed in energy-dispersive X-ray analysis [58]. Furthermore, a preclinical study on albino rats reported its anti-inflammatory activity [59]. A published clinical study on *Muktashukti Bhasma* showed significant improvement in outcome parameters for osteopenia and osteoporosis [57].

Therefore, preliminary evidence suggests that the Ayurveda interventions included in the proposed therapeutic regimen have the potential to reduce the severity of pain and functional disability associated with osteoarthritis. Furthermore, the herbal ingredients of these interventions possess anti-inflammatory, analgesic, antiosteoporotic, antiarthritic, chondroprotective, and antioxidant activities. Therefore, these interventions may alleviate the pathophysiology of osteoarthritis by exerting favorable effects on proinflammatory markers and slowing down the degenerative process.

A well-planned Ayurveda therapeutic regimen grounded in Ayurveda principles and supported by scientific evidence that addresses the needs and challenges of osteoarthritis management and promotes holistic care may significantly improve long-term management. The comprehensive Ayurveda regimen designed for this study has the potential to offer valuable insights into effective therapeutic options for the management of osteoarthritis.

The present RCT protocol has several key strengths. First, it uses a complex Ayurveda regimen that incorporates therapeutic procedures and internal medications, which are easy to administer, well-tolerated, and safe, with the potential to address osteoarthritis-related pathological changes. Second, the study includes an adequate sample size and validated subjective assessment tools, along with imaging and relevant biomarkers as outcomes to address various parameters related to the pathogenesis of osteoarthritis.

### Limitations

This protocol also has a few limitations, including the open-label design of the study. However, to minimize potential bias in assessing the outcomes, the research staff assigned to assess the study outcomes were blinded to the allocation of participants to the study groups. Furthermore, to mitigate the risk of selective reporting of trial results, the statistician who will analyze the study data will also be blinded to the allocation of participants to the study groups.

### Conclusions

The outcomes of the present RCT are expected to generate robust evidence regarding the efficacy and safety of a complex Ayurveda therapeutic regimen in the long-term management of osteoarthritis of the knee, compared with standard conventional care based on validated subjective, laboratory, and imaging parameters. The results and findings will be disseminated following the best practices in scientific publishing, ensuring that the knowledge generated will benefit the scientific community and the public through publications in peer-reviewed, indexed medical journals and presentations at national and international conferences.

## Appendix

Multimedia Appendix 1 Methodology of the therapeutic procedures (Matra Basti and Janu Basti).

## Abbreviations

ACR: American College of Rheumatology
AIIMS: All India Institute of Medical Sciences
CRF: case record form
GCP: Good Clinical Practice
KFT: kidney function test
LFT: liver function test
MRI: magnetic resonance imaging
RCT: randomized controlled trial
SPIRIT: Standard Protocol Items: Recommendations for Interventional Trials
WOMAC: Western Ontario and McMaster Universities Osteoarthritis Index

## Data Availability

The datasets generated or analyzed during this study will be available from the corresponding author on reasonable request.
