# Supplementary material for: Efficacy of a Multimodal Ayurveda Regimen in the Management of Primary Knee Osteoarthritis: Protocol for an Open-Label Randomized Controlled Trial
Source: JMIR Res Protoc. 2025 Sep 3;14:e68306. doi: 10.2196/68306 (PMC12444219; doi:10.2196/68306)
Supplement: Multimedia Appendix 1 [file resprot_v14i1e68306_app1.docx]

**Methodology for the administration of *Matra Basti* and *Janu Basti***

**(as per Guidelines on Basic Training and Safety in Panchakarma published by CCRAS, Ministry of Ayush, Govt. of India)**

***Janu Basti***

The participant will be asked to come on an empty stomach for the therapeutic procedure. Before this procedure, oleation and hot fomentation of the affected knee joint will be done. Warm *Dhanwantara Taila* is poured and left retained in the frame or compartment made of flour of black gram around the knee joint for 30 minutes. Oil is kept warm by repetitively adding hot oil to the frame and replacing the oil at regular intervals. After removing the flour frame, a gentle massage is done, followed by hot fomentation. The same procedure will be followed for 14 days continuously. *Janu Basti* will be administered every two months for six months.

***Matra Basti***

After the *Janu Basti*, participants will be asked to take a light diet, slightly less than the usual quantity. Then, the participant will be advised to take the left lateral position with the left lower extremity straight and the right lower extremity flexed on the knee and hip joint. The anal area will be lubricated with *Ksheerbala Taila* to facilitate the insertion of the catheter. Lukewarm *Ksheerbala Taila* (60 ml) will be taken in a sterile enema syringe. A sterile rubber catheter lubricated with *Taila* will be attached to the enema syringe. The catheter will be introduced (4-5 inches deep) into the anal canal of the participant, avoiding forceful insertion. The participant will be asked to take deep breaths while introducing the catheter and medication. After the administration of *Basti*, the participant will be advised to lie in the supine position. After a while, the participant will be advised to rest. Observe the patient for discomfort, pain, or bleeding during and after the procedure. Dispose of used instruments and materials appropriately to prevent cross-contamination. The same procedure will be followed for 14 days continuously. *Matra Basti* will be administered every two months for six months.

Aseptic precautions will be taken during the entire procedure, such as thoroughly sterilizing all instruments used, carefully cleaning the anal area with an antiseptic solution, wearing gloves by the paramedical staff during the entire procedure, and using a sterile catheter.

**Note:** The therapeutic procedures will not be administered to female participants during menstruation or will be discontinued if they have already started before menstruation. In that case, the procedures may be administered again for the remaining duration after menstruation.
